# Civil society: the catalyst for ensuring health in the age of sustainable development

**DOI:** 10.1186/s12992-016-0178-4

**Published:** 2016-07-16

**Authors:** Julia Smith, Kent Buse, Case Gordon

**Affiliations:** Faculty of Health Sciences Blusson Hall, Simon Fraser University, Room 11802, 8888 University Drive, Burnaby, BC V5A 1S6 Canada; Health Economics and HIV and AIDS Research Division, University of KwaZulu Natal, Durban, South Africa; UNAIDS, 20, Avenue Appia, CH-1211 Geneva 27, Switzerland; IMAXI Cooperative, Cambous, France

**Keywords:** Civil society, Global health, Health policy, Governance, Participation accountability, Sustainable Development Goals, 2030 Agenda for Sustainable Development

## Abstract

Sustainable Development Goal Three is rightly ambitious, but achieving it will require doing global health differently. Among other things, progressive civil society organisations will need to be recognised and supported as vital partners in achieving the necessary transformations. We argue, using illustrative examples, that a robust civil society can fulfill eight essential global health functions. These include producing compelling moral arguments for action, building coalitions beyond the health sector, introducing novel policy alternatives, enhancing the legitimacy of global health initiatives and institutions, strengthening systems for health, enhancing accountability systems, mitigating the commercial determinants of health and ensuring rights-based approaches. Given that civil society activism has catalyzed tremendous progress in global health, there is a need to invest in and support it as a global public good to ensure that the 2030 Agenda for Sustainable Development can be realised.

## The promise and potential of SDG3

The 2030 Agenda for Sustainable Development (the Agenda) promises a profound social transformation with its “indivisible tapestry of thought and action” [1]. Sustainable Development Goal Three (SDG3), to “ensure healthy lives and promote well-being for all at all ages,” is rightly ambitious. Its nine targets represent a significant improvement on the health-related Millennium Development Goals in that they broaden the focus from select diseases to a holistic vision of health – to which everyone is entitled [2]. The breadth and complexity of the goals, as well as the health-related targets in other areas of the Agenda, will however, mean ‘doing’ global health differently. Among other things, progress will require action and coordination beyond the health sector into domains such as trade, addressing commercial drivers of ill health, and strengthening accountability [3]. For these, and other reasons, some argue that the targets are impossible to achieve [4].

Experience illustrates, however, that progress against all odds is possible in global health. Such has been the case most notably on issues with a robust and mobilised civil society. We contend that a progressive civil society is essential to fulfilling eight functions that will be decisive in the quest to achieve the health-related targets in the global agenda. By civil society we refer to voluntary, non-state, not-for-profit organisations formed by people in the social sphere with commonly held values, beliefs and/or causes. Recognising the diversity among civil society organisations (CSOs), we use select examples from progressive ones to demonstrate their catalytic potential for attaining universal health and well-being.

1. **Transforming data into moral arguments**
  While big data, epidemiology and economic modeling are important in agenda setting and policy formulation, leaders are more likely to act on the basis of moral arguments than empirical evidence in response to and in support of generating public sentiment [5]. Civil society, including people most affected by illness and inequities, contributes this emotive force to global health. For example, early AIDS activists demanded world leaders understand the staggering numbers of HIV infections as representing fellow human beings with families and life stories. While politicians and scientists struggled to speak openly about sex, sexuality and drug use, people living with HIV courageously shared their hard-lived experiences of stigma, losing loved ones and failed medical care. They organised die-ins, protested at scientific conferences, and produced art that reflected the unfolding tragedy [6].
  In South Africa, the Treatment Action Campaign, and its allies, publicised stories of people dying from AIDS, thus forcing the government, pharmaceutical corporations and the World Trade Organization, among others, to come face to face with the consequence of their refusal to enable access to affordable AIDS treatment. It was these moral arguments, above the legal or economic, that resulted in 39 major pharmaceutical companies dropping their court case against the government of South Africa, and which lead to the Doha Declaration on the TRIPS Agreement and Public Health in 2001, allowing low and middle income countries greater flexibilities in accessing generic medicines [7]. Advocates for tobacco control, as well as those engaged in the campaign to ban landmines and to establish the Code on the Marketing of Breast Milk Substitutes, have successfully employed similar approaches and tactics [8].
  The power of these moral demands is equally applicable to today’s struggles to address health inequality, access to affordable medicines, road safety, nutritious foods and clean air. However, as the Editor-in-Chief of The Lancet notes, the discourse around many of today’s most pressing health issues lacks this moral urgency. He writes, “The NCD movement is too quiet, too pedestrian, and too polite to make the impact it deserves.... The NCD community needs an electric shock to its semi-comatose soul. But who has the courage to deliver it?” [9]. History indicates that such courage can be found within civil society movements.
2. **Building coalitions that reach beyond the traditional health sector**
  The World Health Organization (WHO) has been lampooned as the World Disease Organization for its lack of engagement with other sectors that determine the risk factors for many of the SDG health targets [10]. Achieving those targets will require new platforms for intersectoral cooperation and incentives to engage in them [11]. Civil society is not confined to ministries or divisions, and so is unrestricted in the partnerships it can form. Tobacco control advocates did not limit themselves to public health alliances. They lobbied the food and hospitality sector to ban smoking in restaurants and bars, worked with ministries of transport to stop smoking on planes, and convinced other CSOs to refuse funding from tobacco companies [12]. Malaysian CSOs, such as the Malaysian Cancer Society, successfully lobbied their government during negotiations over the Trans Pacific Partnership Trade Agreement to table a ‘tobacco carve out’, which will prevent tobacco corporations from using investor state dispute mechanisms to weaken public health measures [13]. The carve out, agreed to by the 12 Pacific Rim countries that signed the agreement in 2016, though an admittedly incomplete public health provision, recognises the relationship between global trade and health, a relationship that will have to be further addressed if SDG3 is to be achieved. In this and countless other areas, civil society provides the impetus for action by building coalitions across multiple sectors, often working with government and other allies, such as scientists or journalists.
3. **Democratising policy debates and offering innovative options**
  Civil society organisations have fought for, and to a degree won, the right to sit at global health governance tables. Civil society delegations to organisations such as the Global Fund to Fight AIDS, Tuberculosis and Malaria (GFATM) and UNAIDS contribute additional voices (often emic), and suggestions drawn from on-the-ground experiences to propose alternative policy options [14]. Delegates to the Global Fund, for example, proposed the creation of the Community System Strengthening Framework and advocated for the Dual Track Financing mechanisms. A broad civil society coalition championed a Framework Convention on Global Health for years before the UN Secretary-General got behind the proposal. Civil society’s meaningful participation has expanded the range of policy-solutions considered, promoting the innovation global health needs to embrace if it is to achieve SDG3 targets.
  At the country level, civil society often advocates for specific policy options once key issues comes onto the political agenda. For example, as the Government of Pakistan reviewed its approach to HIV prevention, HIV service delivery and advocacy organisations, in co-operation with researchers, presented, discussed and reformulated options with government officials so as to improve the chance of their adoption and implementation [15].
4. **Enhancing the legitimacy of global health initiatives and institutions**
  If the engagement of civil society in broadening options under the consideration of policy makers represents the pragmatic side of the civic engagement coin, the other side is the normative one of deepening and extending democracy itself. Given the democratic deficit inherent in global health governance, the meaningful participation of CSOs can contribute to institutional legitimacy [16]. For example, the Global Fund’s insistence on community representation on each national Country Coordinating Mechanism and representation of affected communities on its governance board provides the institution with “normative validity” [17]. This is a legitimacy born from the first-hand experience or the claims of affected people—the power of credible information and moral authority. In such cases, the activist citizen engages not merely in the periodic election of representatives, but throughout the policy cycle or governance process more broadly.
  Deliberative and participatory engagement, as a norm, can lend legitimacy to governance and policy-making bodies, including multilateral ones such as World Health Assembly (WHA). The UN has recognised the legitimising nature of such engagement in terms of strengthening decision-making and outcomes [18]. Numerous case studies attest to civil society organisations acting in this role – from the Ottawa Process to Ban Land Mines [19] to the drafting on the Convention on the Rights of the Child [20], to the UN Convention on the Rights of Person with Disabilities [16]. The social movement #WHO4ALL represents an attempt to open up the deliberations of the World Health Assembly to civic engagement. Participatory engagement represents a critical function of CSOs, but remains underappreciated, underutilised and under-resourced as there is often insufficient policy space—hence the need for social movements to create that space.
5. **Leading the transformation of systems for health fit for 2030 ambitions**
  The Ebola outbreak in West Africa in 2015 highlighted the lethal lack of effective health systems in affected countries, as well as the inadequacy of global co-operation for health [21]. The role of non-governmental organisations (NGOs), such as Médecins Sans Frontiers, in providing emergency public health responses have been much, and rightly, celebrated. Equally important were the activities of community organisations that were able to mobilise local people and resources for a more effective response. In Kailahun, Sierra Leone, the epicenter of the epidemic, the SEND Foundation of West Africa adapted its development programs to train community health workers, organise prevention workshops and provide medical supplies [22]. SEND mobilised local security forces, traditional leaders, faith-based groups and women’s network members to access remote villages that foreign NGOs were unable to reach. After the international panic subsided (and therefore much of the attention and resources) SEND remained, rebuilding its programs and developing initiatives to improve health education and nutrition. Civil society organisations are not only often the first on the ground, they are usually already present, stay beyond the emergency, have a unique ability to harness local resources, and to connect communities to formal health systems. For example, Agypong argues that lessons can be learned from Ghana’s multisectoral cooperative effort in designing more effective responses to infectious disease outbreaks, including Cholera, particularly the manner in which community-based organisations were engaged [23].
6. **Serving as watchdogs and advocates for accountability**
  The ‘follow-up and review’ framework in the 2030 Agenda has been described as “astonishingly vague and timid,” embodying the narrowest conception of state accountability, and neglecting the role of private actors [24]. The perception of weak accountability can only feed pessimistic arguments that bold goals are meaningless when high income countries can renege on commitments, low income countries can claim lack of resources, and private interests can act independently. However, there are also opportunities within the SDGs to develop inclusive accountability frameworks that capitalise on the watchdog functions of CSOs. As Paul Hunt notes, “Because the SDGs are a colossal challenge of extraordinary complexity, they need to be supported by diverse accountability arrangements…It is essential the ‘web of accountability’ includes independent review of stakeholders’ progress, promises and commitments” [25]. CSOs, with their global networks and relative independence, are well positioned to fulfill such review functions—particularly when they work in collaboration with researchers, global health institutions and governments.
  In the area of tobacco and smoking, CSOs have created a unique accountability network around the Framework Convention on Tobacco Control (FCTC). CSOs attend the Conferences of Parties to support states and WHO in improving reporting requirements. Country implementation reports note numerous cases of CSOs assisting with monitoring of Article 5.3 by keeping states abreast of tobacco industry efforts to influence public health policies through front groups and other means [26]. For example, during the negotiations of the 2013 Tobacco Bill in Ghana, an NGO alerted the government that a prominent think tank was acting as a front for tobacco corporations. The government subsequently banned the think tank from further consultation [27].
  Where CSOs feel states are not adequately reporting on implementation of the FCTC, they produce shadow reports that, among other functions, motivate states to improve future reporting [28]. And, when there are notable transgressions, CSOs use creative sanctions such as the Golden Ash Tray awards, which are given to governments, corporations and media entities that fail to implement tobacco control measures. Tobacco control CSOs have created a web that functions to both support states and institutions in developing accountability systems, and acts as a watchdog.
  Similar approaches have been fought for in the global response to AIDS. Civil society participates in UNAIDS working groups to ensure monitoring indicators reflect the needs and experiences of service delivery organisations and key populations. At the national level, CSOs partner with states to produce annual Country Progress Reports, and publish shadow reports when necessary. Reporting on progress toward global commitments to AIDS is considered one of the most effective models of non-binding instruments in global health policy, in part a function of CSO engagement [29]. The success of the SDGs will arguably hinge on the implementation of similarly inclusive participatory approaches to policy development, monitoring and review.
7. **Demanding action to address commercial determinants of (ill) health**
  The ambitions embedded in SDG3 raise questions about how to protect health when trade agreements grant harmful industries access to markets around the world, decisions made in a corporate office in one country affect health outcomes in another, and the commercial marketing of harmful substances pervasively crosses borders [30]. Civil society activism provides one answer.
  In Honduras’ export processing zones, garment workers suffer from neck and spinal injuries from hunching over machines for long workdays. CODEMUH, a women’s labour organisation, demands that government enforce labour laws which require factory owners to accommodate injured workers, challenges companies that discriminate against workers with injuries, and assists workers in filing complaints [31]. CODEMUH won two cases at Honduras' Supreme Court in 2015, is appealing four cases involving women who were wrongfully dismissed from their jobs, and is proposing a new occupational health and safety law. The combined strategy of advocating for stronger state legislation, while putting pressure on corporations to comply with regulations, has enabled CODEMUH to improve the health of young women. Similar strategies by CSOs can prompt states to impose regulations to protect workers from hazardous chemicals, and to protect the broader public from pollution and other harmful products and processes.
  Aiming to address threats posed by the commercial junk food industry, CSOs have called for a binding health treaty, similar to the FCTC, to tackle poor diets. The World Obesity Federation, Consumers International and UK Health Forum write, in a letter to the Director Generals of the FAO and WHO that “the governance of food production and distribution cannot be left to economic interests alone” [32]. Instead they advocate action on the commercial determinants of poor diets such as: regulations to reduce children’s exposure to marketing, compositional limits on saturated fat, sugar and sodium content; and that all trade an investment policies be assessed for their potential health impacts. At the national level, consumer movements in Latin America have convinced some governments to institute junk food taxes [33]. By proposing and campaigning for such legislation, civil society creates political incentives and policy options for leaders to take action to protect public health, as opposed to furthering commercial interests alone.
8. **Ensuring the right to health is universally enjoyed by all**
  The SDGs are celebrated for the language of universality – for addressing development challenges in all countries, not just lower-income contexts. However, there is no explicit mention of the need for human rights-based approaches (HRBA) to attaining the health targets in SDG3, despite ample evidence that such approaches are essential to achieving universal goals [34]. While it can be hoped that the emphasis on universal rights in the 2030 Agenda preamble will be brought to bear on each of the goals, history suggests that it is civil society that will ensure that human rights are upheld [35].
  Indeed it is CSOs that have fought for the right to health against all odds. In Afghanistan organisations such as the Afghan Women’s Educational Center and Shuhada Organisation have fought for women’s human rights, including to health, since the 1980s [36]. These organisations not only provide healthcare to women and children throughout wars and political oppression, but also advocate for civil and political rights, and implement economic empowerment programs. These programs demonstrate that the human right to health is indivisible from other rights, and essential to peaceful development; a principle reflected in the preamble to 2030 Agenda, and which must be asserted to achieve SDG3.

## Conclusion

We do not romanticize civil society. We recognise that not all organisations and activists have progressive aims. As was recently pointed out, there are “uncivil” organisations, as well as those that work towards rights, equality and development [37]. We further appreciate that pro-poor health policy reform typically requires broad alliances built between national and international officials, researchers, journalists and others.

Nor do we advocate CSOs replacing the essential functions of states in ensuring the right to health through acting on its determinants and ensuring access to services. Indeed many of the examples above, such as ensuring the tobacco carve out within the TPP, involve advocacy coalitions through which CSOs work with states and other non-state actors to achieve transformations in health. In other cases, such as in Honduras, CSOs provide motivation for states to strengthen their role in governing health. We suggest CSOs’ particular characteristics, such as independence and links to populations most affected, position them as essential partners in fulfilling specific functions. Given that civil society activism has catalyzed tremendous progress in global health, we consider civic engagement as vital to the transformation promised by the SDGs.

We recognise the need for further research on role of CSOs in health governance at national and global levels [38]. Much existing research either idealises or rejects the positive influence of civil society [39]. What is needed is research that contextualises specific civil society engagements within health governance partnerships and arrangements, testing theoretical assumptions, and analysing outcomes of CSO engagement. The eight functions we identify here could provide a framework for further empirical analysis.

There is also a need to document the contemporary challenges CSOs around the world face in participating in health governance and service delivery. The space for civil society is shrinking; increased surveillance to combat security threats is restricting, at times purposefully and at times by default, the freedom of speech that enables CSOs to act as watchdogs and advocates for the marginalised. States around the world are implementing legislation to prohibit what are portrayed as foreign organisations, but are in fact global civil society movements driven by local people [40]. Many of the leading civil society organisations in global health, as well as those providing direct services, are struggling for survival, due to decreased resources. This trend will have to be reversed if SDG3 is to be achieved. The historic commitment to finance civil society, made in the 2016 UN Political Declaration on Ending AIDS, recognises both the essential functions CSOs fulfill and the need to support them in doing so [41].

While much energy, anger and urgency will remain the prerogative of ‘outsider’ civil society watchdog organisations, much more effort needs to be made to provide civil society a place at decision-making tables as envisioned in SDG 16 on inclusive societies. As respected partners, there is need to support CSOs’ organisation, participation, empowerment, mobilisation and advocacy by investing in the sector as the global public good it is if we are to achieve health and well-being by 2030.

## Abbreviations

CSOs, civil society organisations; FCTC, Framework Convention on Tobacco Control; GFATM, Global Fund to Fight AIDS, Tuberculosis and Malaria; HRBA, human rights-based approaches; NCD, non-communicable disease; NGO, non-governmental organization; SDG, sustainable development goal; TRIPS, trade related aspects of intellectual property rights; UNAIDS, United Nations Joint Program on AIDS; WHO, World Health Organization
